# Editorial: New techniques for modelling, prognosis, diagnosis, and treatment of human knee pathology

**DOI:** 10.3389/fbioe.2024.1412924

**Published:** 2024-05-14

**Authors:** Abdelwahed Barkaoui, Bernardo Innocenti, João Manuel R. S. Tavares

**Affiliations:** ^1^ Université Internationale de Rabat, Sale, Morocco; ^2^ Université Libre de Bruxelles, Brussels, Belgium; ^3^ Faculdade de Engenharia, Universidade do Porto, Porto, Portugal

**Keywords:** knee, biomechancis, diagnostic, pronostic, pathologies and risk prevention

Pathologies of the human knee represent a significant challenge for healthcare professionals, affecting individuals’ quality of life and incurring substantial costs for healthcare systems. These conditions can result from various factors, such as sports injuries, age-related wear and tear, or underlying medical conditions. Over recent years, significant advancements in the modelling, prognosis, diagnosis, and treatment of these knee pathologies have been driven by technological progress and a better understanding of knee physiology and biomechanics. These advancements have led to the development of more precise and personalized techniques to improve clinical outcomes and patient recovery. By combining state-of-the-art medical imaging, biomechanical analysis, and innovative therapeutic approaches, healthcare professionals are better equipped than ever to diagnose and effectively treat knee pathologies, enabling patients to regain optimal functionality and improved quality of life.

The guest editors are delighted to present this Research Topic of Frontiers in Bioengineering and Biotechnology - Biomechanics, dedicated to “New Techniques for Modelling, Prognosis, Diagnosis, and Treatment of Human Knee Pathology”, which brings together a diverse range of expertise to explore the challenges and opportunities in the complex field of knee biomechanics.

The knee, one of the most essential and complex joints in the human body, faces various challenges, notably osteoarthritis, a degenerative disease that can devastate quality of life. With a growing prevalence of knee osteoarthritis and its significant socioeconomic consequences, it is imperative to develop innovative approaches for early detection, prevention, and effective treatment of this condition.

Under this perspective, this Research Topic focuses on the latest advances in the field, highlighting progress in modelling, prognosis, diagnosis, and treatment of knee pathologies. The 11 contributions from authors representing five countries (Spain, China, United Kingdom, Germany, and Chile), give a comprehensive understanding of the field from different angles.

These contributions encompass several Research Topic, including the influence of tension-band plates on mechanical loading and their effects on the femoral growth plate, alongside motion capture’s role in detecting kinematic abnormalities in osteoarthritis patients. Additionally, studies delve into foot biomechanics using dual fluoroscopic imaging, the impact of patellar morphology on knee gait, ligament laxity, and finite element analysis of posterior tibial plate fixation. Further investigations compare machine learning and deep learning for predicting knee biomechanics, explore tibial shock attenuation with non-Newtonian fluid material footwear, and introduce intraoperative sensor technology in the knee domain.

The included contributions can be summarized as:

• Wang et al. investigated markerless motion capture’s effectiveness in identifying kinematic abnormalities in knee osteoarthritis (KOA) during the Functional Movement Screen (FMS) and daily activities, suggesting the FMS, particularly knee flexion and trunk angles during in-line lunge, as potential indicators for KOA assessment.
• Peng et al. examined knee joint loading during badminton lunges at various distances and foot positions using dual fluoroscopic imaging, revealing increased knee flexion and ground reaction forces with longer lunges, and larger knee translation and torque with externally rotated foot positions, indicating potential injury risks.
• Wang et al. explored the relationship between patella morphology, transverse alignment, and knee gait in healthy Chinese adults over 40, finding no significant association between patella morphology/transverse alignment and knee gait parameters, but highlighting the femoral-tibial angle’s influence on the knee adduction moment.
• Wu et al. assessed knee laxity in patients with ACL injury using a digital arthrometer, indicating significantly lower stiffness in the ACL injury group than controls, particularly in later loading stages, suggesting potential diagnostic value for knee laxity assessment.
• Hu et al. aimed to improve the fixation of posterolateral tibial plateau fractures (PTPF) using various screw fixation methods, with biomechanical tests showing superior strength in two-screw fixation compared to single-screw fixation, supported by finite element analysis.
• Shao et al. compared non-Newtonian (NN) shoes with ethylene vinyl acetate (EVA) shoes in cushioning and reducing sports injuries, revealing NN shoes’ superior cushioning during exercise, particularly in high-temperature conditions.
• Stoddart et al. examined different knee arthroplasty techniques’ effects on bone load transfer, indicating higher strain shielding with total knee arthroplasty (TKA) compared to partial knee arthroplasty (PKA) and potential long-term bone health implications.
• Zhang et al. compared deep learning with conventional machine learning methods in predicting knee biomechanics post-total knee arthroplasty, demonstrating deep learning’s superior accuracy and potential for precise alignment assessment.
• Valente et al. investigated the impact of tibiofemoral alignment and contact point locations on knee contact forces in individuals with varus malalignment, highlighting contact points’ significant influence on knee forces during different activities.
• Ge et al. assessed inter-prosthetic pressures post-Oxford unicompartmental knee arthroplasty and their correlation with lower limb alignment, revealing correlations between pressures at specific knee angles and postoperative alignment parameters.
• Hucke et al. studied the mechanical influence of tension-band plates used in correcting knee malalignment through guided growth therapy, showing heterogeneous stress distribution in the growth plate, with implants inducing static stress in the insertion region and altering cyclic loading, affecting growth rates, with potential implications for preventing malalignment recurrence.

These articles illustrate the diversity of approaches and methodologies used to address the complex challenges of knee pathologies from various angles, including mechanical, biomedical, digital, and experimental. They thus underscore the growing importance of research in this domain. The Research Topic addressed in this Research Topic go beyond mere academic research; they contribute significantly to understanding the biomechanical behavior of the knee joint and its pathologies, particularly in early detection and prediction of knee osteoarthritis.

In conclusion, this Research Topic provides a captivating overview of recent progress in the field of knee biomechanics. The guest editors thank the editors, authors, and reviewers for contributing to this stimulating Research Topic, hope this Research Topic will inspire new ideas, collaborations, and advancements in the fight against knee pathologies, and look forward to future developments in this domain.

